# Development and Application of Human Renal Proximal Tubule Epithelial Cells for Assessment of Compound Toxicity

**DOI:** 10.2174/2213988501711010019

**Published:** 2017-02-14

**Authors:** Shuaizhang Li, Jinghua Zhao, Ruili Huang, Toni Steiner, Maureen Bourner, Michael Mitchell, David C. Thompson, Bin Zhao, Menghang Xia

**Affiliations:** 1 9800 Medical Center Drive, National Center for Advancing Translational Sciences, National Institutes of Health, Bethesda, MD 20892-3375, USA; 2 Sigma-Aldrich Corporation, St. Louis, MO 63103, USA; 3 Research Center for Eco-Environmental Sciences, Chinese Academy of Sciences, Beijing 10085, China

**Keywords:** Apoptosis, Mitochondrial membrane potential (MMP), Nephrotoxicity, SA7K cells, Transporters

## Abstract

Kidney toxicity is a major problem both in drug development and clinical settings. It is difficult to predict nephrotoxicity in part because of the lack of appropriate *in vitro* cell models, limited endpoints, and the observation that the activity of membrane transporters which plays important roles in nephrotoxicity by affecting the pharmacokinetic profile of drugs is often not taken into account. We developed a new cell model using pseudo-immortalized human primary renal proximal tubule epithelial cells. This cell line (SA7K) was characterized by the presence of proximal tubule cell markers as well as several functional properties, including transporter activity and response to a few well-characterized nephrotoxicants. We subsequently evaluated a group of potential nephrotoxic compounds in SA7K cells and compared them to a commonly used human immortalized kidney cell line (HK-2). Cells were treated with test compounds and three endpoints were analyzed, including cell viability, apoptosis and mitochondrial membrane potential. The results showed that most of the known nephrotoxic compounds could be detected in one or more of these endpoints. There were sensitivity differences in response to several of the chemicals between HK-2 and SA7K cells, which may relate to differences in expressions of key transporters or other components of nephrotoxicity pathways. Our data suggest that SA7K cells appear as promising for the early detection of renal toxicants.

## INTRODUCTION

Humans are exposed to a variety of drugs, natural products, industrial chemicals and environmental pollutants that can induce nephrotoxicity, leading to acute renal failure. Twenty percent of the episodes of acquired acute renal failure in the community and hospitals settings are caused by drugs [1-3]. The mechanisms underlying nephrotoxicity are complex and include tubular cell toxicity, altered intraglomerular hemodynamics, crystal nephropathy, inflammation, thrombotic microangiopathy and rhabdomyolysis [4-6]. Animal models have the limitations of high-cost, low-throughput and sometimes inconsistent prediction of human toxicity [7]. *In vitro* cell-based assays that are target-specific and mechanism-oriented represent a promising alternative to animal toxicology studies [8]. There is still a great need for the development of new *in vitro* cell models for evaluating and predicting drug or chemical-induced nephrotoxicity.

Proximal tubule cells are a common site of drug-induced kidney injury due to their exposure to circulating chemicals and their role in transporter-mediated drug clearance [9]. Tubular cell toxicity is commonly caused by impaired tubular transport, mitochondrial dysfunction, increased oxidative stress and production of free radicals [6, 10]. There are numerous transporters expressed on both the basolateral and apical membranes of the renal tubule epithelium, which systematically mediate renal elimination of drugs. The basolateral membrane transporters include both the organic anion (*e.g.* OAT1, OAT3, OATP4C1) and the organic cation transporter families (*e.g.* OCT2), which mediate the uptake of anionic and cationic drugs from the blood into proximal tubule cells. Apical membrane transporters function both to secrete drugs into the urine and to reabsorb compounds from the urine back into the proximal tubule cells. These transporters include the multidrug and toxin extrusion proteins (MATE1, MATE2-K), the multidrug resistance protein 1 (MDR1, P-glycoprotein or P-gp), multidrug resistance-associated proteins (MRP2, MRP4), the oligopeptide transporters (PEPT1, PEPT2), additional organic anion and cation transporters (OAT4, OCTN1, OCTN2) and a urate transporter (URAT1). Localization, expression, substrates and inhibitors of these renal drug transporters have been reviewed [11]. Several known nephrotoxicants, such as cephalosporin, cisplatin, adefovir dipivoxil, cyclosporine A and ifosfamide, have been shown to be directly transported by or interact with renal transporters [12-15].

Several *in vitro* cell models have been developed and used for nephrotoxicity evaluation. However, there are no validated or regulatory approved *in vitro* models available for predicting nephrotoxicity [16]. Human embryonic kidney 293 (HEK293), porcine kidney (LLC-PK1), human kidney-2 (HK-2), and hRPTEC/TERT1 are cell lines that have been previously used in nephrotoxicity evaluation [17-19]. More recently, human induced pluripotent stem cell-derived renal cells have been used to study nephrotoxicity [20]. Most of these cell-based models have not been fully characterized for relevant transporter expression nor have they been reported to have activity for some key drug transporters. For example, in HK-2 cells, the expression of uptake transporters (OAT1, OAT3 and OCT2) was not detected and the expression of the apical efflux transporters (P-gp, MRPs) was low relative to human cortical tissue levels [21]. The RPTEC/TERT1 cell line expressed relevant transporters at both the mRNA and protein levels [22], but the establishment of functional transport assays was not successful with this cell line [16]. Immortalized cell lines are reported to be less sensitive than human primary renal proximal tubular cells and insensitive to well-known nephrotoxicants [23], which may be due in part to changes in drug transporter expression associated with immortalization [24]. Therefore, interest remains high in finding or developing better *in vitro* renal cell model systems.

An alternative approach to generating kidney cell lines involves targeting cell cycle proteins in order to enable bypass of cellular senescence. Here we report the generation of a human kidney proximal tubule cell line (SA7K) *via* zinc finger nuclease-mediated knockout of a cell cycle protein. This pseudo-immortalized cell line had extended cell doubling capacity and was characterized in terms of kidney-specific functional properties, such as response to a limited number of known human nephrotoxicants, as well as uptake and efflux transporter activities. Results from these preliminary studies prompted us to consider whether SA7K cells were suitable for higher throughput screening assays for nephrotoxicity. For this purpose, the current study was designed to evaluate the response of SA7K cells to a group of nephrotoxic compounds. Cell viability, apoptosis and mitochondrial membrane potential, were assessed in both SA7K and the human kidney HK-2 cells. The HK-2 cell line was used as a comparator cell line.

## MATERIALS AND METHODS

### Compounds

Test compounds used for the assays were identified from the literature. All test compounds and reagents were obtained from Sigma-Aldrich (St. Louis, MO) unless specified otherwise. Each compound was prepared and serially diluted in DMSO in 1536-well microplates to yield 11 concentrations. The final concentration of the 34 well-characterized compounds related to nephrotoxicity and 10 compounds showing renal toxicity in the 5 μL assay volume ranged from 31.2 nM to 184 μM and 0.7 nM to 46 μM, respectively.

### Generation of SA7K Cells and Cell Culture

Human primary kidney proximal tubule epithelial cells were obtained from Zen-Bio Inc. (Research Triangle Park, NC). Zinc finger nuclease (ZFN) pairs were delivered into the SA7K cells by nucleofection. Cells were split into two pools, one for population doubling studies and the other for FACS sorting (single-cell isolation). Cells were sorted based on negative selection for carboxyfluorescein succinimidyl ester (CFSE), a dye that distinguishes fast growing cells from slow growing ones. Cells that did not have detectable CFSE were single-cell sorted into 96-well plates. Clones with the desired mutation were identified initially by fragmentation analysis and later confirmed by sequencing of the targeted area. SA7K cells were cultured in RPTEC Complete Supplement medium while nephrotoxicity experiments were carried out using RPTEC Tox Supplement medium (Sigma-Aldrich). HK-2 cells were purchased from ATCC (Manassas, VA) and cultured in keratinocyte growth medium (Lonza Inc, Mapleton, IL).

### Characterization of SA7K Cells

#### Immunocytochemical (ICC) Staining

Cells were plated on 4 well culture slides at 0.125×10^6^ cells/well for 2 days. Cells were stained with anti-CD13 (ab7417, mouse monoclonal WM15 from Abcam, Cambridge, MA) for 1 hour at room temperature. Cells were rinsed with Tris buffered saline with Tween 20 (TBST) and then incubated with Donkey Anti –Mouse FITC antibody from Jackson ImmunoResearch (West Grove, PA) for 1 hour. Cells were rinsed with TBST and imaged.

#### Gamma-glutamyl transpeptidase (GGT) Activity

GGT activity was measured by following the release of para nitroanilide (pNA) from gamma glutamyl-p-nitroanilide using glutamyltransferase (GGT) activity colorimetric assay kit from Sigma-Aldrich. HPTC (1x10^6^) or SA7K cells (1x10^6^) were homogenized in 200 µL of ice-cold GGT assay buffer. Ten µL of these samples combined with 90 µL of GGT substrate solution were added to assay plate. Absorbance changes (418 nm) of the assay plate were measured every 20 minutes at 37 °C for 2 hours in incubation.

#### Response to Parathyroid Hormone (PTH)

PTH was obtained from Prospec (New Brunswick, NJ). After overnight incubation with 0.1 mM IBMX (3-isobutyl-1-methylxanthine, phosphodiesterase inhibitor), cells were treated with 1-1000 nM PTH for 15 or 30 minutes. Intracellular cyclic AMP (cAMP) was measured using cAMP direct EIA kit from Arbor Assays (Ann Arbor, MI).

#### Albumin Uptake

FITC-labeled albumin was added to cell incubations and was then incubated for 15, 30 and 60 minutes at 4°C or 37°C. Cells were washed 8 times with ice cold Ringers Solution (pH 7.3) and lysed with 0.1% Triton X100 in 1X MOPS. Fluorescence was measured at 485/520 nm.

### Measurement of Transporter Expression

HPTC, SA7K and HK-2 cells were seeded on 24-well plates at 0.125×10^6^ cells/well and cultured for 3 days prior to RNA extraction (RNeasy Protect Mini Kit, Qiagen, Valencia, CA). Total extracted RNA was quantified and purity verified using the Thermo Scientific NanoDrop 2000 Spectrophotometer. RNA (150 ng) were analyzed per reaction, in triplicate, using the Applied Biosystems’ TaqMan^®^ RNA-to-CT™ 1-step kit in MicroAmp^®^ optical 96-well reactions plates. GAPDH was used as the negative control. Taqman^®^ gene expression assay probes were acquired from Thermo Fisher Scientific (Waltham, MA).

### Transporter Assay

SA7K cells were seeded on BD Falcon 24-well plates at 0.5×10^6^ cells/well for 2 days. For uptake assays (OAT1 and OCT2), cells were pre-incubated with inhibitor (probenecid or doxepin, where indicated) for 10 min at 37°C. Substrates (p-aminohippuric acid (PAH) for OAT1, amantadine for OCT2) were added and incubated for another 10 minutes at 37°C. The reactions were stopped with ice-cold buffer followed by washing 3 times with ice-cold HBSS. Samples were extracted with 250 μL methanol and analyzed by liquid chromatography–tandem mass spectrometry (LC/MS/MS). For efflux assays (P-gp and MRP2), cells were pre-incubated with inhibitor (verapamil or MK571, where indicated) for 30 minutes at 37°C, then incubated with substrate with or without the inhibitor for another 10 minutes. Substrates used were SN-38 for MRP2 and digoxin for P-gp. Samples were extracted with 250 μL methanol and dried under nitrogen. Samples were resuspended in 125 µL of 50% acetonitrile and analyzed by LC-MS/MS.

### Cell Viability Assay

The cell viability of SA7K and HK-2 cells was measured using a CellTiter-Glo Luminescent Cell Viability Assay (Promega, Madison, WI) after compound treatment. The change of intracellular ATP content indicates the number of metabolically competent cells. The SA7K and HK-2 cells were seeded at 2,000 cells/5 μL/well in 1536-well white, solid bottom plates (Greiner Bio-One, North America, NC) using a Multidrop Combi (Thermo Fisher Scientific, Waltham, MA). After the cells were incubated at 37°C for 5 hours, 23 nL of compounds at 11 concentrations was transferred to the assay plates by a Pintool station (Kalypsys, San Diego, CA). DMSO was used as a vehicle control and tetraoctylammonium bromide was used as a positive control. For the 15 well-known compounds the plates were incubated for 48 hours at 37°C, followed by the addition of 5 μL per well of CellTiter-Glo reagent. After 30 minutes of incubation in dark at room temperature, the luminescence intensity was measured using a ViewLux plate reader (PerkinElmer, Shelton, CT).

### Caspase 3/7Assay

Caspase activity was measured in SA7K and HK-2 cells after 24 h compound treatment using a Caspase-Glo 3/7 Assay kit (Promega, Madison, WI). The SA7K and HK-2 cells were dispensed in assay medium at 2,000 cells/5 μL/well in 1536-well white plates using a Multidrop Combi. After the cells were incubated at 37°C for 5 hours, 23 nL of compounds was added *via* the Pintool station. Staurosporine, a caspase inducer, was used as a positive control. Treated cells were incubated for 24 hours at 37°C, followed by the addition of 5 μL per well of Caspase-Glo 3/7 reagent. After 30 minutes of incubation at room temperature, the luminescence intensity of the plates was measured using a ViewLux plate reader.

### Mitochondrial Membrane Potential Assay

The Mitochondrial Membrane Potential Assay (Codex Biosolutions, Montgomery Village, MD) is a fluorescence-based assay that quantifies the mitochondrial membrane potential changes. The SA7K and HK-2 cells were dispensed at 2000 cells/5 µL/well in 1536-well black clear-bottom assay plates. The assay plates were incubated at 37°C overnight before 23 nL of compound was transferred from the compound plate to the assay plate *via* a Pintool station. The assay plates were incubated for 1 hour at 37°C, followed by the addition of 5 μL/well of dye-loading solution. After the assay plates were incubated at 37°C for 30 minutes, fluorescence intensity at 490 nm excitation and 535 nm emission wavelengths were measured using an EnVision plate reader (PerkinElmer, Shelton, CT). Data were expressed as the ratio of 590 nm/535 nm emissions.

### Data Analysis

Data were analyzed as described previously [25, 26]. Briefly, data were normalized to DMSO controls (0%) and the maximal response of positive control compounds (100% or - 100%) and corrected by applying a NCATS in house pattern correction algorithm [27]. Concentration response data for each compound were fitted to the Hill equation, yielding concentrations of half-maximal effective or inhibitory concentration (EC_50_/IC_50_) and maximal response (efficacy).

## RESULTS

### Generation and Characterization of SA7K Cell Line

**Fig. (1):**
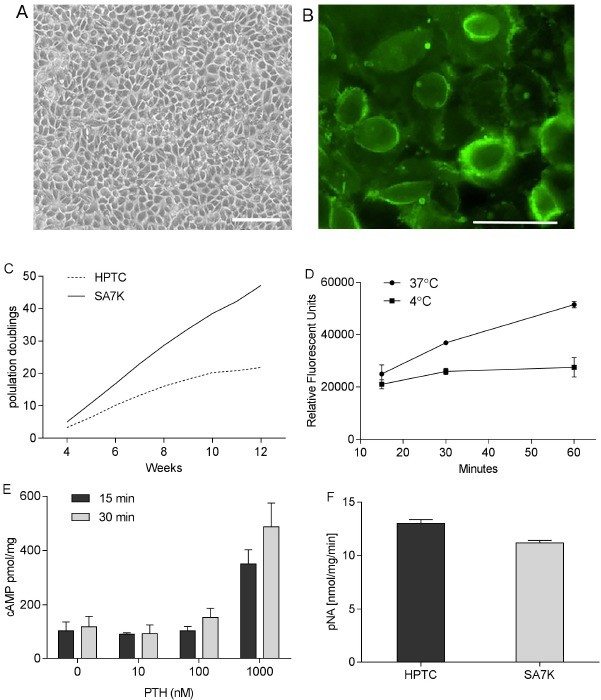
Growth, morphological and functional characterization of SA7K cells. (**A**) Normal epithelial morphology of SA7K cells, including dome formation; (**B**) Expression of the proximal tubule marker aminopeptidase N (CD13) in SA7K cells; (**C**) Extended population doublings in ZFN-modified SA7K *versus* HPTC cells; (**D**) Uptake of Albumin-FITC; (**E**) Response to Parathyroid Hormone (PTH); (**F**) GGT Activity in HPTC *versus* SA7K cells. Scale bar: 50 µm.

In the present study, we modified human primary renal proximal tubule epithelial cells (HPTC) by knocking out a cell cycle regulatory protein using ZFN technology, thereby enabling extended cell proliferation. The resulting clones demonstrated an extended population doubling capacity compared with HPTC (passage 2) as illustrated in Fig. (**1A**). SA7K clone was selected based primarily on growth and morphological characterization. This clone exhibited normal epithelial morphology including dome formation, which is indicative of transepithelial solute transport (Fig. **1B**). The proximal tubule origin of the cells was confirmed by expression of the transmembrane biomarker aminopeptidase N (CD13) as shown in Fig. (**1C**). Several additional proximal tubule specific functional properties were also evaluated in the SA7K cells. Albumin resorption from the urinary filtrate is thought to occur *via* cubilin/megalin receptors located on the apical membrane of proximal tubule cells [28]. SA7K cells demonstrated time- and temperature-dependent albumin uptake using FITC-labeled albumin (Fig. **1D**). Proximal tubules are known to respond to the parathyroid hormone (PTH) by increasing calcium resorption and phosphate secretion through a cAMP-dependent mechanism [29]. Exposure of SA7K cells to the PTH resulted in a concentration- and time-dependent increase in cAMP, suggesting PTH receptor expression (Fig. **1E**). *Gamma* glutamyl transpeptidase (GGT) is an enzyme located on the brush border (apical) surface of proximal tubules [30]. GGT activity was detected by monitoring cleavage of *gamma* glutamyl-*p*-nitroanilide and was comparable to HPTC (Fig. **1F**).

### Transporter Expression in SA7K Cells

**Fig. (2):**
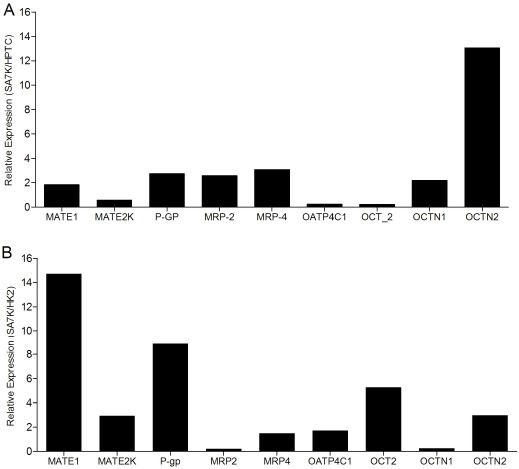
Expression profile of transporters. (**A**) HPTC *versus* SA7K cells; (**B**) SA7K *versus* HK-2 cells. The mRNA levels of nine known proximal tubule transporters were measured by RT-PCR in HPTC, SA7K and HK-2 cells.

Transporters play an important role in the uptake/secretion mechanisms of proximal tubule cells, and may contribute to elevated, potentially toxic intracellular concentration of drugs or metabolites. As noted above, immortalized renal cell lines have been reported to have low or non-detectable levels of one or more drug transporters [21]. Therefore, the mRNA expression levels of the major uptake and efflux drug transporters were measured in SA7K and HK-2 cells (Fig. **2**). Nine transporters were detected at various levels in the SA7K cells, with the notable exception of OAT1 and OAT3. The highest levels of expression were observed for P-gp, MRP4, OATP4C1 OCTN1 and OCTN2 (threshold cycle, CT, values ranging from 21-26), while lower levels were observed for MATE1, MATE2-K, MRP2 and OCT2 (CT values from 27-32). Seven out of the nine transporters measured were higher in SA7K cells than HK-2 (Fig. **2B**) . In addition, the expression profile of uptake and efflux transporters in SA7K cells was similar to that of the early passage human primary donor cells used as source material.

### Functional Activity of Transporters in SA7K Cells

**Fig. (3):**
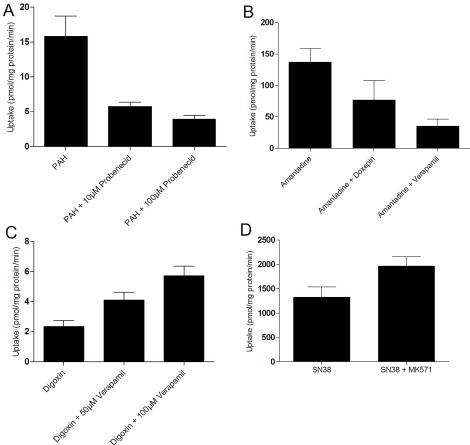
Functional Characterization of Uptake and Efflux Transporter Activity in SA7K cells. (**A**) Uptake of PAH by OAT-1 and inhibition by probenecid; (**B**) Uptake of amantadine by OCT2 and inhibition by doxepin; (**C**) Inhibition of MDR1-mediated efflux of digoxin by verapamil; (**D**) Inhibition of MRP2-mediated efflux of SN-38 by MK571. Substrate concentrations used were 10 µM in each experiment.

It is well-known that mRNA levels do not always correlate with transporter protein levels or activity [31]. To functionally assess the transporters, we measured the activities of two uptake and two efflux transporters. Uptake of the OCT2 substrate amantadine and the OAT1 substrate PAH were readily observed in SA7K cells (Fig. **3A, B**). While doxepin (10 µM) inhibited amantadine uptake by 46%, probenecid inhibited PAH uptake by 64% and 78% at concentrations of 10 µM and 100 µM, respectively. Interestingly, OAT-dependent uptake was observed in spite of undetectable mRNA levels. The activity of the OATs was highly dependent on the culture conditions used. For the efflux transporters, P-gp-mediated efflux of digoxin was inhibited with 50 and 100 µM verapamil, leading to a 1.8-fold and 2.5-fold increase in intracellular digoxin, respectively (Fig. **3C**). Similarly, MRP2-mediated efflux of SN-38 was inhibited using 10 µM MK571, leading to a 50% increase in intracellular SN-38 (Fig. **3D**). Thus, SA7K cells maintained functional activity for both uptake and efflux transporters, suggesting their potential use in studying drug-transporter interactions and evaluating renal toxicants.

### Assessment of Well-characterized Nephrotoxicants

To evaluate the nephrotoxicity, SA7K and HK-2 cells were both exposed to a group of 34 well-characterized compounds, 15 of which are known to be toxic to proximal tubule cells, 10 of which are not directly toxic for PT cells and nine of which are non-nephrotoxic compounds (20); all 34 compounds were used to treat cells at various concentrations ranging from 31.2 nM to 184 µM. After exposure, cell viability, cell apoptosis and mitochondrial membrane potential (MMP) were determined. To determine cell viability, the endpoint for evaluating cytotoxicity was the measurement of cellular ATP content. Caspase 3/7 induction in cells is an indicator of cell apoptosis, which can be employed to evaluate the toxicity of compounds. Mitochondrial damage and dysfunction are known to be closely related to kidney diseases [32]. Therefore, mitochondrial function represents an initial step in predicting exposure-related toxicity of compounds that potentially reduce the mitochondrial membrane potential (MMP) [26]. Out of the 15 nephrotoxicants tested, 10 showed nephrotoxic effects in the three endpoints tested (Table **1**). The other five compounds including gentamicin, 5-fluorouracil, tobramycin, ifosfamide and tenofovir were negative in our assays (data not shown); however, the reported IC_50_s of these 5 compounds were much higher than the highest concentration used in our study [20]. Among the 10 positive compounds confirmed in our study, eight and seven compounds showed cytotoxic effects in HK-2 and SA7K cells respectively. In the caspase 3/7 and MMP assays, SA7K cells showed higher sensitivity than HK-2 cells, and the three endpoints were complementary in predicting nephrotoxicity. The other 19 compounds were inactive (supplemental Table **1**) in viability and caspase 3/7 assays except atorvastatin and triiodothyronine; atorvastatin showed cytotoxicity and apoptosis induction in both SA7K cells and HK-2 cells and triiodothyronine showed cytotoxicity only in HK-2 cells. Moreover, four compounds out of 19, including triiodothyronine, atorvastatin, ibuprofen and lindane decreased MMP in SA7K and HK-2 cells, indicating good specificity of SA7K cells. Ibuprofen and lindane are nephrotoxic compounds that are not directly toxic to PT cells, implicating their induction of renal toxicity may occur through the mitochondria related pathway. Therefore, MMP assays might be a good endpoint through which to evaluate nephrotoxicity. Thus, multiple assays employing cell viability, cell apoptosis and MMP may be a promising avenue for early detection of nephrotoxicity for compounds with a high potency.

### Evaluation of Other Related Nephrotoxic Compounds in HK-2 and SA7K Cells

**Fig. (4):**
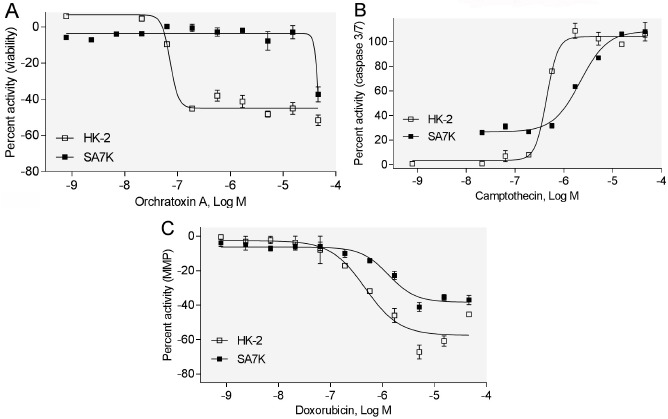
Concentration response curves of selected compounds in HK-2 and SA7K cells. (**A**) Effect of ochratoxin A on cell viability; (**B**) Effect of campothecin on caspase 3/7; (**C**) Effect of doxorubicin on MMP.

To further evaluate SA7K cells, we tested 10 more compounds, which have been reported to be associated with renal dysfunction, including camptothecin [33], sunitinib malate [34], sulfinpyrazone [35], mitomycin C [36], vinblastine sulfate [37], doxorubicin [38], taxol [39], ochratoxin A [40], ergotamine [41], and digoxin [42]. The potencies of these compounds are listed in Table **2**. Among these 10 compounds tested in the viability assay, seven compounds displayed toxicity utilizing the HK-2 cells, while six compounds showed toxicity in the SA7K cells. In the caspase 3/7 assay, five compounds exhibited an increase in caspase 3/7 activity when treating the HK-2 cells, whereas six compounds showed an increase in this endpoint in the SA7K cells. In the MMP assay, five compounds decreased MMP in both HK-2 and SA7K cells. With respect to the IC_50_ values, there were several compounds that showed differences in sensitivity between HK-2 and SA7K. For example, Ochratoxin A, camptothecin, and doxorubicin showed different dose-response curves in HK-2 and SA7K cells (Fig. **4A-C**). These different responses may be related to differences in expression of the renal transporters or other related components of nephrotoxicity pathways.

## DISCUSSION

In this study, we developed and validated an *in vitro* cell model (SA7K) for nephrotoxicity prediction. The cell line was created from human primary proximal tubule cells by ZFN-mediated knock out of a key cell cycle protein, thus enabling extended population doublings. Renal drug transporters play an important role in the absorption, elimination, metabolism, and toxicity of many prescribed drugs. The SA7K cells maintained similar expression level of several uptake and efflux transporters as well as CYP3A4 when compared to human primary proximal tubule cells at early passages. In addition, transporting functionality of uptake and efflux transporters was observed. Cell viability, Caspase 3/7 and mitochondrial membrane potential were used as endpoints to identify nephrotoxicity in a 1536-well plate format. The mRNA levels of OAT1 and OAT3 from the current study were not detected, however, it has been reported that gene expression of OAT1 and OAT3 was rapidly lost in other cell culture experiments [43]. Interestingly, OAT-dependent uptake was observed in our study and the reason for this discrepancy remains to be clarified.

The data presented here identified a difference in compound responses when comparing SA7K and HK-2 cells. HK-2 cells have been reported to lack the function of several uptake and efflux transporters [21]. Transporters are important for the uptake/secretion mechanisms of proximal tubule cells, affecting the intracellular concentration of drugs or metabolites that play a vital role in the toxicity of numerous compounds. OAT1 and OCT2, expressed on the basolateral membrane of proximal tubule cells, are responsible for transporting the anionic and cationic small molecules into the epithelial cells, while P-gp and MRP2 can protect cells by excreting potentially toxic xenobiotics [44]. Camptothecin is a substrate of both P-gp and MRP2 [45], while doxorubicin is a substrate of P-gp [46]. Therefore, camptothecin and doxorubicin may accumulate at increased levels and have a higher potency in HK-2 cells. Ochratoxin A can be transported into the cell by OAT1 and OAT3, while also being secreted by MRP2 and BCRP [47]. The different response ochratoxin A generated between HK-2 and SA7K remains to be elucidated. Therefore, transporters should be considered in the development of *in vitro* cell models for nephrotoxicity prediction. In addition, the role of transporters in drug-induced nephrotoxicity still needs further study.

SA7K cells performed well when identifying the well-characterized and reported nephrotoxic compounds. Compared with the HK-2 cells, SA7K cells were able to detect a similar percentage of known and suspected nephrotoxicants and displayed specificity in this regard. Compared with a single endpoint assay, our assay which employs three endpoints was more sensitive, and therefore is amenable for high throughput screening. Measuring MMP is considered as the first step in predicting exposure-related toxic effects [26]. Therefore, the MMP assay was first applied to predict nephrotoxicity in our study and indicated high sensitivity. Many known nephrotoxicants that did not show activity in our assay had higher IC_50_s values than the concentrations we used in the current study. For example, the reported IC_50_s of gentamicin, 5-fluorouracil, tobramycin, ifosfamide and tenofovir are at millimolar levels [20], but in our 1536-well screens, compounds were only tested from nanomolar to micromolar level.

In summary, SA7K cells maintained proximal tubule cell markers as well as several functional properties. The presence of functional uptake and efflux transporters coupled with toxicant sensitivity suggest that this cell line may be useful for drug-transporter interactions and for the early detection of renal toxicants.

## Figures and Tables

**Table 1:** Cytotoxicity of well-characterized human nephrotoxicants in HK-2 and SA7K Cells.

**Nephrotoxicants**	**Cell viability (IC_50_, µM)**		**Caspase 3/7 (EC_50_, µM)**		**MMP (IC_50_, µM)**	
	HK-2	SA7K	HK-2	SA7K	HK-2	SA7K
Cyclosporine A	19.92±4.17	Inactive	Inactive	11.12±1.80	Inactive	10.34±1.86
Potassium dichromate	65.75±14.83	18.65±5.27	Inactive	23.39±1.90	Inactive	72.54±4.91
Cadmium (II) chloride	26.98±4.60	65.23±11.73	Inactive	Inactive	Inactive	Inactive
Citrinin	78.2±0	87.75±0	Inactive	98.46± 0	78.56±9.03	73.96±6.01
Adefovir dipivoxil	64.65±4.38	91.71±11.69	63.23±14.46	84.57±5.51	Inactive	78.21±0
Puromycin	1.30±0.64	3.95±0.64	3.37±0.22	2.55±0.82	Inactive	5.28±2.68
Arsenic trioxide	13.15±1.07	17.19±4.29	Inactive	15.16±6.0	Inactive	Inactive
Rifampin	84.57±5.51	69.70±0	Inactive	82.98±6.75	26.89±3.70	15.67±5.70
Tacrolimus	Inactive	Inactive	Inactive	Inactive	49.93±0	22.30±0
Tetracycline	Inactive	Inactive	Inactive	Inactive	Inactive	9.85±0

Data are presented as mean ± SD from two to three experiments. Compounds showing no concentration response were defined as inactive.

**Table 2:** Evaluation of nephrotoxic compounds in HK-2 and SA7K Cells.

**Compounds**	**Cell viability (IC_50_, µM)**		**Caspase 3/7 (EC_50_, µM)**		**MMP (IC_50_, µM)**	
	HK-2	SA7K	HK-2	SA7K	HK-2	SA7K
Camptothecin	0.69±0.09	0.75±0.14	0.47±0.05	1.92±0.54	6.26±0.51	14.54±3.63
Sunitinib malate	18.03±3.09	12.65±3.06	Inactive	7.33±1.78	Inactive	Inactive
Sulfinpyrazone	22.21±1.81	Inactive	Inactive	Inactive	14.82±1.28	11±4.15
Mitomycin C	10.11±0.66	7.53±2.90	23.79±4.44	6.38±0.42	Inactive	Inactive
Vinblastine Sulfate	Inactive	Inactive	Inactive	0.57±1.86	Inactive	Inactive
Doxorubicin	3.89±0.50	2.51±0.74	2.64±0.79	2.75±0.35	0.39±0.19	1.18±0.51
Taxol	Inactive	Inactive	0.04±0.01	0.67±0.29	34.08±4.45	35.87±2.34
Ochratoxin A	0.04±0	17.00±1.38	0.15±0.06	Inactive	Inactive	Inactive
Ergotamine	Inactive	Inactive	Inactive	Inactive	17.64±1.43	16.16±2.75
Digoxin	0.03±0.008	0.61±0.19	Inactive	Inactive	Inactive	Inactive

Data are presented as mean ± SD from two to three experiments.
